# Supplementary material for: Molecular characterization of breast cancer cell response to metabolic drugs
Source: Oncotarget. 2018 Jan 8;9(11):9645–60. doi: 10.18632/oncotarget.24047 (PMC5839391; doi:10.18632/oncotarget.24047)
Supplement: Supplementary file 1 [file oncotarget-09-9645-s001.pdf]

Molecular characterization of breast cancer cell response to metabolic drugs

SUPPLEMENTARY MATERIALS

Supplementary Table 1: SNP array. See Supplementary_Table_1

Supplementary Table 2: Protein data. See Supplementary_Table_2

Supplementary Table 3: MTF delta values. See Supplementary_Table_3

Supplementary Table 4: RP delta values. See Supplementary_Table_4

Supplementary Table 5: Prediction of MTF response using proteomics data

Protein	B	Std. error	Beta	Sig
**MMGT1**	120.295	0.342	1.070	0.000
**IDH1**	8.233	0.118	0.208	0.000
**PSPC1**	2.559	0.109	0.068	0.000
**TACO1**	−1.279	0.129	-0.033	0.000

Linear regression model that predicts MTF response (adjusted $R^2 = 1$) using protein expression data. B = constant, Std. error = standard error, Beta = covariate, Sig = statistical significance.

Supplementary Table 6: Prediction of RP response using proteomics data

Protein	B	Std. error	Beta	Sig
**ACADSB**	−14.894	0.154	−0.838	0.000
**CCDC58**	7.248	0.208	0.330	0.000
**MPZL1**	−1.837	0.105	−0.094	0.000
**SBSN**	−1.945	0.307	−0.079	0.000

Linear regression model that predicts RP treatment response (adjusted $R^2 = 1$) using proteomics data. B = constant, Std. error = standard error, Beta = covariate, Sig = statistical significance.

Supplementary Table 7: Prediction of RP response using functional data

Model	B	Std. error	Beta	Sig
**Constant**	1.095	0.075		0.001
**Metabolism A**	−2.171	0.282	−0.960	0.005
**Metabolism B**	−1.149	0.356	−0.403	0.048

Multiple linear regression model predicting response against RP treatment. B = constant, Std. error = standard error, Beta = covariate, Sig = statistical significance.

Supplementary Table 8: Cytometry experiments. See Supplementary_Table_8

Supplementary Table 9: FBA and FVA results. See Supplementary_Table_9

Supplementary Table 10: Prediction of MTF response using flux data

Model	B	Std. error	Beta	Sig
**Constant**	0.779	0.002		
**Glutamate metabolism**	−2.379	0.016	−0.848	0.004
**Pyruvate metabolism**	0.083	0.002	0.224	0.016

Linear regression model using flux activities to predict MTF response. B = constant, Std. error = standard error, Beta = covariate, Sig = statistical significance.

Supplementary Table 11: Prediction of RP response using flux data

Model	B	Std. error	Beta	Sig
**Constant**	0.761	0.001		0.000
**Valine, leucine and isoleucine metabolism**	2.018	0.002	0.929	0.001
**Cholesterol metabolism**	0.045	0.000	0.093	0.007

Linear regression model using flux activities to predict RP response. B = constant, Std. error = standard error, Beta = covariate, Sig = statistical significance.

Supplementary Table 12: Catalase and superoxide dismutase delta values between control and MTF-treated cell lines

Cell line	Catalase	Superoxide dismutase
**MCF7**	0.36	0.31
**T47D**	0.18	1.3
**CAMA1**	−0.15	1.25
**MDAMB231**	0.11	0.45
**MDAMB468**	0.20	0.09
**HCC1143**	0.39	0.90

Delta was calculated, subtracting MTF catalase and superoxide dismutase cell fluxes to control cell fluxes.

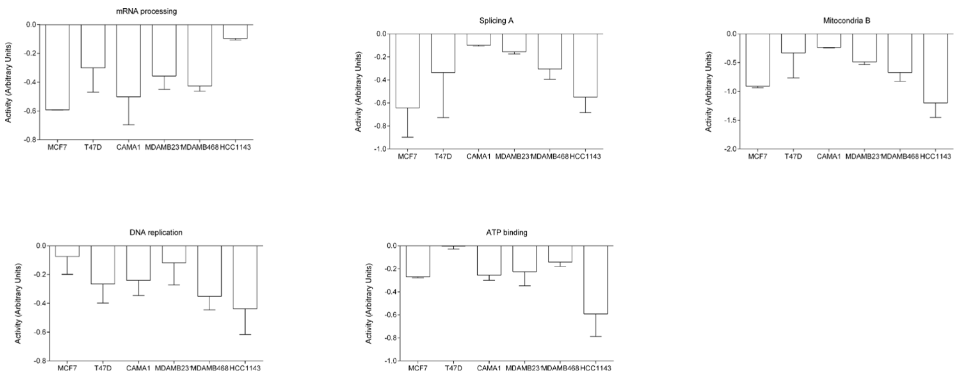

Supplementary Figure 1: Node activity measurements for cell lines treated with MTF with a decrease in functional node activity in all cell lines.

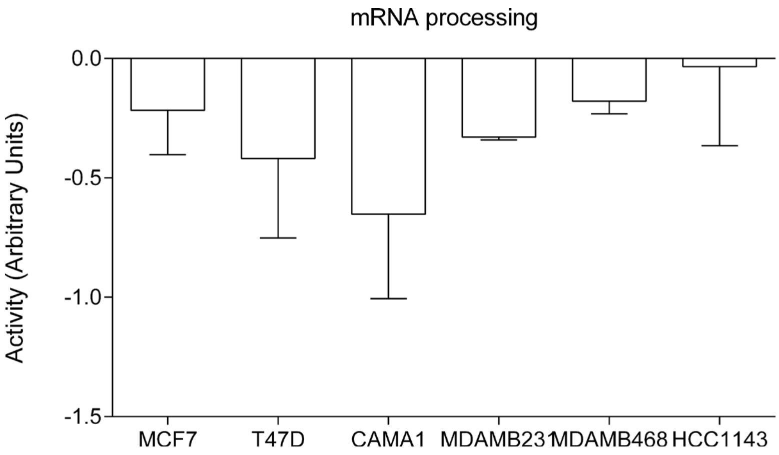

Supplementary Figure 2: Node activity measurements for cell lines treated with RP with a decrease in functional node activity in all cell lines.

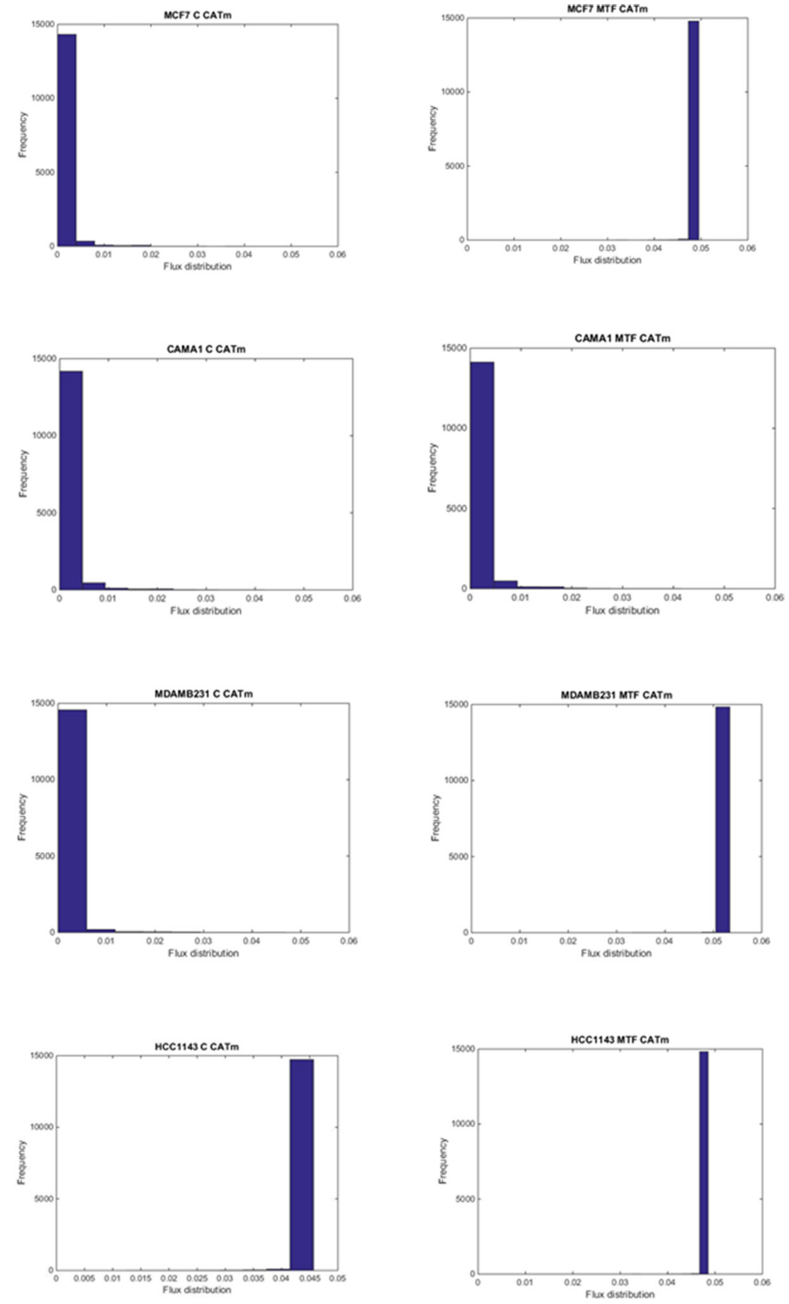

Supplementary Figure 3: Distribution of possible fluxes of catalase (CATm) in control and MTF cell cultures. On the y-axis, frequency is represented; understood as number of possible flux combinations provided by Monte Carlo resampling.

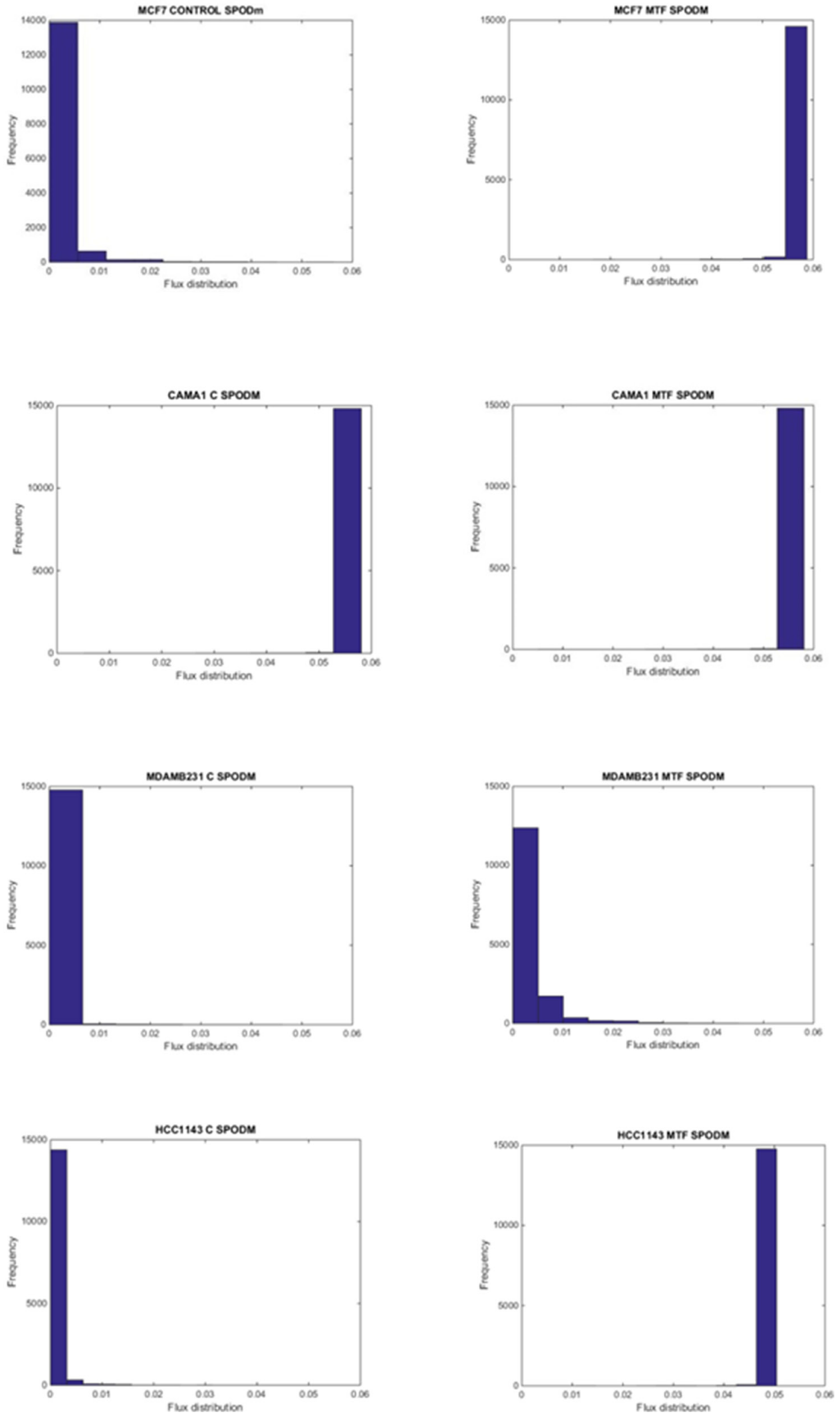

Supplementary Figure 4: Distribution of possible fluxes of superoxide dismutase (SPODM) in control and MTF cell cultures. On the y- axis, frequency is represented; understood as number of possible flux combinations provided by Monte Carlo resampling.

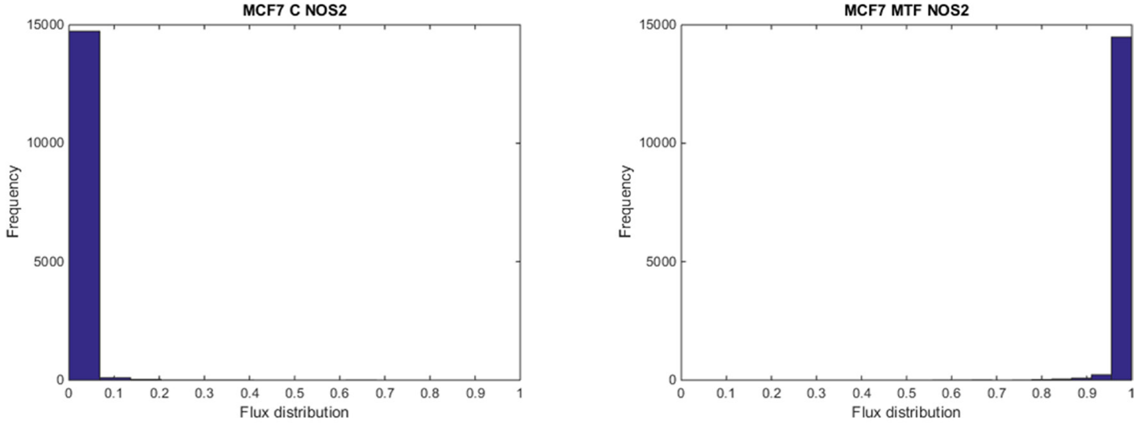

Supplementary Figure 5: Distribution of possible fluxes of nitric oxide synthase (NOS2) in control and MTF MCF7 cell cultures. The model predicts an increase in NO in MTF-treated MCF7 cells regarding the control. On the y- axis, frequency is represented; understood as number of possible flux combinations provided by Monte Carlo resampling.

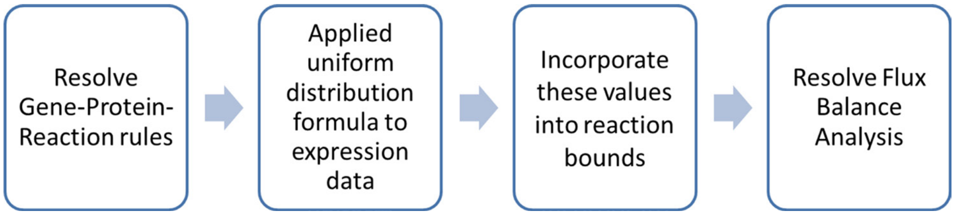

Supplementary Figure 6: E-flux flowchart.

Supplementary Files 1–12: Monte Carlo sampling for each cell line and condition. (S1 File: MCF7 Control 1, S2 File: MCF7 MTF 1, S3 File: MCF7 RP 1, S4 File: CAMA1 Control 1, S5 File: CAMA1 MTF 1, S6 File: CAMA1 RP 1, S7 File: MDAMB231 Control 1, S8 File: MDAMB231 MTF 1, S9 File: MDAMB231 RP 1, S10 File: HCC1143 Control 1, S11 File: HCC1143 MTF 1, S12 File: HCC1143 RP 1).

Supplementary File 13: Code to incorporate normalized protein expression data into the model. This data needs to be previously normalized using modified E-flux. See Supplementary_File_13
